# Detoxification, Active Uptake, and Intracellular Accumulation of Chromium Species by a Methane-Oxidizing Bacterium

**DOI:** 10.1128/AEM.00947-20

**Published:** 2021-01-04

**Authors:** Salaheldeen Enbaia, Abdurrahman Eswayah, Nicole Hondow, Philip H. E. Gardiner, Thomas J. Smith

**Affiliations:** a Biomolecular Sciences Research Centre, Sheffield Hallam University, Sheffield, United Kingdom; b Biotechnology Research Centre, Tripoli, Libya; c School of Chemical and Process Engineering, University of Leeds, Leeds, United Kingdom; Shanghai Jiao Tong University

**Keywords:** *Methylococcus*, bioavailability, bioremediation, heavy metals, methanotrophs

## Abstract

Despite the wide-ranging proscription of hexavalent chromium, chromium(VI) remains among the major polluting heavy metals worldwide. Aerobic methane-oxidizing bacteria are widespread environmental microorganisms that can perform diverse reactions using methane as the feedstock. The methanotroph Methylococcus capsulatus Bath, like many other microorganisms, detoxifies chromium(VI) by reduction to chromium(III). Here, the interaction of chromium species with *M. capsulatus* Bath was examined in detail by using a range of techniques. Cell fractionation and high-performance liquid chromatography–inductively coupled plasma mass spectrometry (HPLC–ICP-MS) indicated that externally provided chromium(VI) underwent reduction and was then taken up into the cytoplasmic and membranous fractions of the cells. This was confirmed by X-ray photoelectron spectroscopy (XPS) of intact cultures that indicated negligible chromium on the surfaces of or outside the cells. Distribution of chromium and other elements within intact and sectioned cells, as observed via transmission electron microscopy (TEM) combined with energy-dispersive X-ray spectroscopy (EDX) and electron energy loss spectroscopy (EELS), was consistent with the cytoplasm/membrane location of the chromium(III), possibly as chromium phosphate. The cells could also take up chromium(III) directly from the medium in a metabolism-dependent fashion and accumulate it. These results indicate a novel pattern of interaction with chromium species distinct from that observed previously with other microorganisms. They also suggest that *M. capsulatus* and similar methanotrophs may contribute directly to chromium(VI) reduction and accumulation in mixed communities of microorganisms that are able to perform methane-driven remediation of chromium(VI).

**IMPORTANCE** *M. capsulatus* Bath is a well-characterized aerobic methane-oxidizing bacterium that has become a model system for biotechnological development of methanotrophs to perform useful reactions for environmental cleanup and for making valuable chemicals and biological products using methane gas. Interest in such technology has increased recently owing to increasing availability of low-cost methane from fossil and biological sources. Here, it is demonstrated that this versatile methanotroph can reduce the toxic contaminating heavy metal chromium(VI) to the less toxic form chromium(III) while accumulating the chromium(III) within the cells. This is expected to diminish the bioavailability of the chromium and make it less likely to be reoxidized to chromium(VI). Thus, *M. capsulatus* has the capacity to perform methane-driven remediation of chromium-contaminated water and other materials and to accumulate the chromium in the low-toxicity chromium(III) form within the cells.

## Abstract (precis)

*M. capsulatus* Bath is a well-characterized aerobic methane-oxidizing bacterium that has become a model system for biotechnological development of methanotrophs to perform useful reactions for environmental cleanup and for making valuable chemicals and biological products using methane gas. Interest in such technology has increased recently owing to increasing availability of low-cost methane from fossil and biological sources. Here, it is demonstrated that this versatile methanotroph can reduce the toxic contaminating heavy metal chromium(VI) to the less toxic form chromium(III) while accumulating the chromium(III) within the cells. This is expected to diminish the bioavailability of the chromium and make it less likely to be reoxidized to chromium(VI). Thus, *M. capsulatus* has the capacity to perform methane-driven remediation of chromium-contaminated water and other materials and to accumulate the chromium in the low-toxicity chromium(III) form within the cells.

## INTRODUCTION

Despite worldwide regulation of the use of hexavalent chromium in industry, this highly toxic, and bioavailable form of chromium continues to be a substantial environmental problem. Hence, environmental microorganisms that can detoxify and sequester chromium species are of biotechnological interest. Despite a fall in commercial use of hexavalent chromium *per se*, various forms of chromium are heavily used in industry, especially in the production of chromium-iron alloys such as stainless steel and in leather manufacture, in which chromium(III) salts are used. Mining of chromite, the major chromium-containing ore, also causes substantial environmental release of chromium species. Such anthropogenic contamination contains significant hexavalent chromium, which may leach into the aqueous environment (1, 2). Chromium(III), from natural or human sources, may be solubilized by organic acids produced by living systems and reoxidized in the soil under oxic conditions, particularly in the presence of manganese compounds that can mediate O_2_-driven oxidation of chromium(III) to chromium(VI) (3–5). Siderophores, which naturally act as microbial iron-scavenging molecules, can also bind and solubilize chromium(III) (6). In most jurisdictions, the maximum contaminant level (MCL) for chromium(VI) in drinking water has not been fixed, though 10 μg liter^−1^, which was in force in California between 2014 and 2017, has been used as a benchmark of the safe level of chromium(VI) (7). Substantial concentrations of Cr(VI) are found in groundwater, for example, up to 1 mg liter^−1^ in certain areas of California (7) and up to 34 μg liter^−1^ in drinking water wells in the Piedmont area of North Carolina (8). The particulate fraction of the exhaust from biodiesel combustion may contain up to 2 mg/g of chromium (9). Worldwide, waste materials and contaminated sites requiring remediation have reported chromium(VI) concentrations up to tens of parts per million (10, 11).

Many bacteria can bioremediate chromium(VI) via reduction to the less harmful trivalent form (12). The metabolic diversity of prokaryotes provides a wide range of natural and artificial electron donors for chromium(VI) reduction (12, 13). Among these, methane is particularly attractive because it is available in large quantities from fossil sources and biogas. It has aroused greater interest in recent years due to falling methane prices (14). Aerobic methanotrophs, a diverse group of environmental bacteria that are able to use methane as their carbon and energy source, are significant as a global methane sink. Methanotrophs and their enzymes have been explored for a range of biotechnologically valuable methane-driven processes, including bioremediation and production of single-cell protein, and as catalysts for oxygenation of unfunctionalized carbon atoms in organic molecules (15–19).

The gammaproteobacterial methanotroph Methylococcus capsulatus Bath is able to reduce chromium(VI) to chromium(III) over a wide range of concentrations (tested across 1.4 to 1,000 mg liter^−1^) (20). Methane-driven chromium(VI) reduction has also been achieved in a methane-fed polymicrobial biofilm reactor system (21), where some of the reduction of Cr(VI) is attributed to nonmethanotrophs scavenging nutrients (multicarbon compounds and more generally accessible C_1_ substrates such as methanol) produced by the methanotrophs.

Methanotrophs are known to bind and transform a range of toxic metals and metalloids in addition to chromium. The alphaproteobacterial methanotrophs, such as Methylosinus trichosporium OB3b, produce a range of structurally related copper-scavenging molecules termed methanobactins (22). Methanobactin-bound copper is in the +1 oxidation state; binding of Cu(II) to methanobactin results in its reduction to Cu(I) (23), possibly by electrons derived from water (22). Methanobactin is able to bind a wide range of cations; a subset of these, including Hg(II) and Au(III), undergo reduction upon binding, in a similar manner to copper, to give metallic mercury and metallic gold nanoparticles, respectively (24–27). Methanobactin binds methylmercury (MeHg^+^); in *M. trichosporium*, methanobactin is necessary for detoxification of MeHg^+^ as well as promoting *in vivo* methylation of Hg(II) (24, 27, 28). The gammaproteobacterial methanotroph *M. capsulatus* Bath reduces Hg^2+^ ions to metallic mercury (29) and takes up but does not detoxify MeHg^+^. Methanotrophs respond to lanthanide elements that are required for activity of a key metabolic methanol dehydrogenase (25, 30, 31). They also convert selenite (SeO_3_^2-^) to selenium-containing nanoparticles and volatile methylated selenium species (32, 33).

Previous work on remediation of Cr(VI) by *M. capsulatus* Bath showed that chromium(III) accumulated in the particulate fraction of the culture and (based on extended X-ray absorption spectroscopy fine structure [EXAFS] results) was likely coordinated by oxygen and phosphorus (20). Previously, electron microscopy techniques coupled with spectroscopic analysis have been used with other systems to characterize the distribution and speciation of chromium associated with bacteria at the cellular or subcellular level (21, 34–37). Here, we used a range of cell fractionation, analytical, electron-microscopic, and spectroscopic techniques to obtain spatially resolved information about the interaction of chromium species with *M. capsulatus*, to determine how its specific properties might be exploited for bioremediation and to gain insights into the role such methanotrophs may play in the environmental chromium cycle.

## RESULTS

### Effect of concentration on chromium(VI) removal.

**FIG 1:**
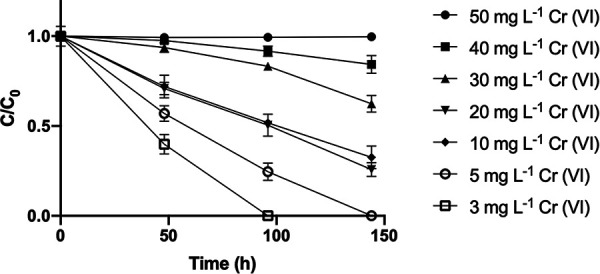
Effect of *M. capsulatus* Bath cultures on Cr(VI) at various concentrations. Experiments were biological triplicates. Results are plotted as the ratio of supernatant chromium(VI) concentration at each time point (*C*) to initial concentration (*C*_0_), and data are means and standard deviations (SD). Parallel triplicate controls without *M. capsulatus* Bath cells were performed at each initial chromium(VI) concentration, which were constant to within 4% of the initial chromium(VI) concentration.

High-performance liquid chromatography (HPLC)–inductively coupled plasma mass spectrometry (HPLC–ICP-MS) is a well-established technique for quantifying and determining the speciation of heavy metals and other elements that has been previously used to characterize reduction of chromium(VI) at a bulk culture level (for example, see reference 38). In order to characterize the range of chromium(VI) concentrations over which *M. capsulatus* could remediate all or most of the added chromium(VI), various concentrations of chromium(VI) were added to cultures of *M. capsulatus* Bath (optical density at 600 nm [OD_600_] of 0.7 to 0.9), and then the cultures were incubated at 45°C in the presence of methane and air. Chromium species in the culture supernatant were quantified by using HPLC–ICP-MS (Fig. 1). *M. capsulatus* Bath achieved complete removal of detectable chromium(VI) at initial concentrations up to 5 mg liter^−1^ and was able to substantially decrease the chromium(VI) of the culture supernatant from initial concentrations of ≤40 mg liter^−1^. No other detectable chromium species appeared in the culture supernatant. A concentration of chromium(VI) of 20 mg liter^−1^ was chosen for further experiments, since this concentration resulted in the largest amount of chromium(VI) removed within 144 h.

### Reduction and accumulation of chromium species within cellular fractions.

**FIG 2:**
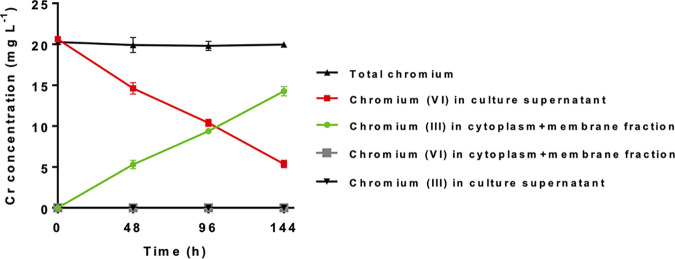
Reduction and accumulation of chromium species by *M. capsulatus* Bath after addition of Cr(VI) to 20 mg liter^−1^. Values are the means from biological triplicates and SD. Concentrations in each of the fractions were normalized to the volume of the original culture.

In order to gain more information about the possible location of chromium species within the culture, cultures were incubated with an initial concentration of chromium(VI) of 20 mg liter^−1^, and samples were taken over a period of 144 h. Cells within each sample were then broken and separated into cell walls and a combined fraction of membranes and cytoplasm, as detailed in Materials and Methods, before quantification of the chromium species via HPLC–ICP-MS (Fig. 2).

Over a period of 144 h, the concentration of chromium(VI) in the culture supernatant declined, while there was a corresponding increase in the concentration of chromium(III) in the cytoplasm-membrane fraction. No other chromium species were detected at significant concentrations in any of the samples. The constant total chromium (the sum of the chromium detected in all fractions) (Fig. 2) indicates that the appearance of chromium(III) in the cytoplasm-membrane fraction accounted, within experimental error, for the decrease in chromium(VI) in the culture supernatant. Hence, cells of *M. capsulatus* Bath not only were able to reduce chromium(VI) to chromium(III) but also appeared to be able to accumulate all the chromium(III) within the biomass.

**FIG 3:**
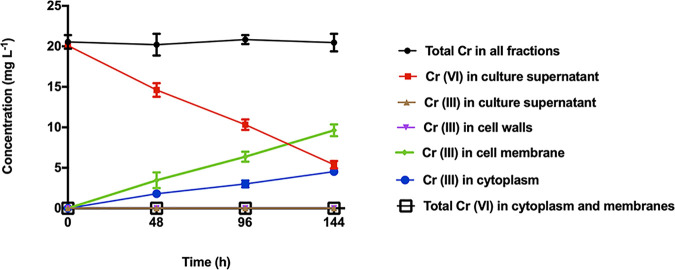
Speciation and distribution of chromium species analyzed after fractionation of cells into separate cell wall, cytoplasm, and membrane fractions. The initial Cr(VI) concentration was 20 mg liter^−1^. Error bars show the standard deviations for three biological replicates.

To gain additional information about the location of chromium species within the cells, cultures were exposed to chromium(VI) (20 mg liter^−1^), and cells were fractionated to produce separate membrane and cytoplasm fractions. The results showed that the distribution of chromium(III) between the two fractions was approximately two-thirds in the cell membrane fraction and one-third in the cytoplasm fraction (Fig. 3).

### Uptake of Cr(III).

**FIG 4:**
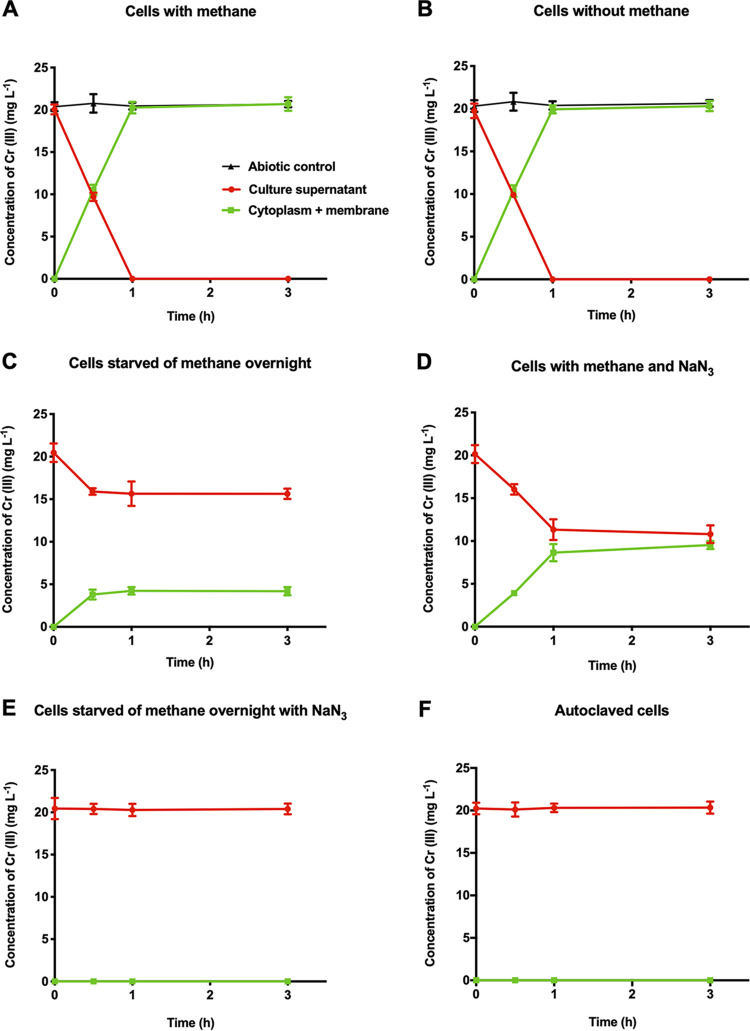
Effect of adding 20 mg liter^−1^ of Cr(III) to *M. capsulatus* Bath cultures with and without methane. The abiotic controls were culture medium plus methane (A) and culture medium without methane (B).

The fact that all of the cell-associated chromium was in the +3 oxidation state, even though the cells had been exposed to chromium in the hexavalent form, raised the question of whether reduction and uptake of chromium were necessarily linked or whether cells could take up trivalent chromium directly. When exposed in exactly the same way to 20 mg liter^−1^ of chromium(III) in the presence of methane and air, the *M. capsulatus* cells took up the chromium(III) completely into the cytoplasm-membrane fraction within 1 h (Fig. 4A), much more quickly than the >144 h taken for reduction and accumulation of the same amount of chromium(VI). Previous work has shown that the reduction of chromium(VI) to chromium(III) by *M. capsulatus* Bath is an active process requiring the presence of the carbon and energy source methane (20). In order to investigate whether the uptake of chromium(III) was also an active process, cultures were exposed to 20 mg liter^−1^ of chromium(III) aerobically but in the absence of methane. When the cells were grown to an OD_600_ of 0.7 to 0.9 in the presence of methane and then methane was removed and chromium(III) added immediately, all the chromium(III) was taken up by the cells within 1 h (Fig. 4B). If, however, the cells were starved of methane overnight (16 h) before addition of the chromium(III), only 25% of the chromium(III) was taken up (Fig. 4C). Addition of the metabolic inhibitor sodium azide to 0.05% (wt/vol) (Fig. 4D) abolished approximately half of the uptake of chromium(III) within a 1-h period. When the cells were starved of methane overnight and sodium azide was added at the same time as the chromium(III), uptake of chromium(III) was completely abolished (Fig. 4E). Heat killing (autoclaving) of the cells also completely abolished chromium(III) uptake (Fig. 4F). These results indicate that uptake of chromium(III) is an active process but also that when methane is removed from a growing culture, it has sufficient reserves of energy to take up a substantial amount of chromium(III).

### Spatially resolved spectroscopic characterization of cells.

**FIG 5:**
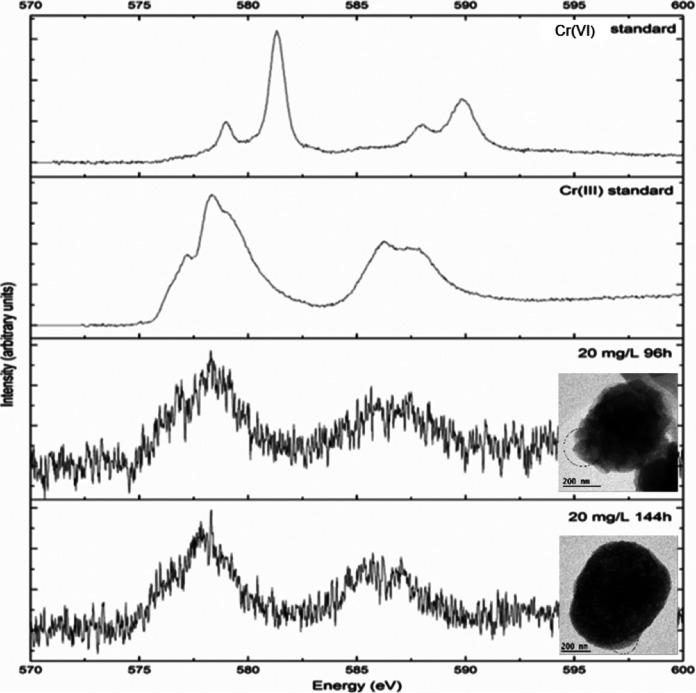
EEL spectra of *M. capsulatus* Bath cells compared with chromium standards. Insets show the areas of the samples (circled) that were analyzed by EELS. Initial Cr(VI) concentration was 20 mg liter^−1^.

**FIG 6:**
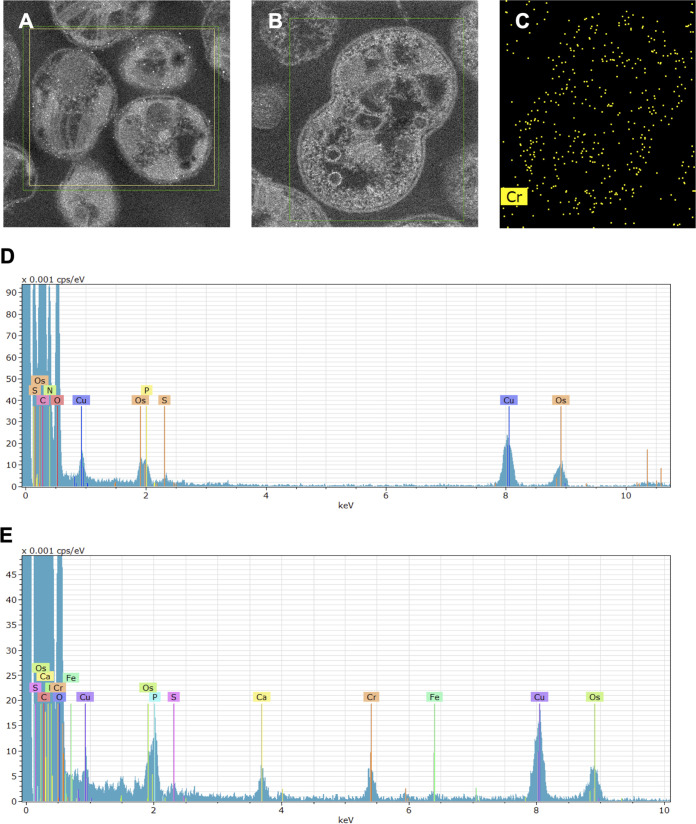
HAADF-STEM and EDX of sectioned cells showing the distribution of chromium. (A) HAADF images of cells without exposure to chromium; (B) HAADF images of cells exposed to 20 mg liter^−1^ chromium(VI) for 144 h; (C) spatial distribution of chromium in the EDX map of the sample shown in B. Green and yellow boxes on the micrographs in panels A and B show the areas of the sample analyzed in the EDX spectra of the samples without (D) and with (E) exposure to chromium(VI).

Electron energy loss spectroscopy (EELS) coupled with TEM of whole *M. capsulatus* Bath cells exposed to 20 mg liter^−1^ of chromium(VI) for 96 h or 144 h confirmed, via comparison with spectra of chromium standards, that the cell-associated chromium was in the +3 oxidation state (Fig. 5). Cells exposed to 20 mg liter^−1^ of chromium(VI) for 144 h were also prepared as thin sections to see how chromium and other elements were distributed within the cells. High-angle annular dark-field (HAADF) scanning transmission electron microscopy (STEM)–energy-dispersive X-ray spectroscopy (EDX) showed the presence of chromium in the chromium-treated cells and its absence from the chromium-untreated control (Fig. 6). The spatial distribution of chromium (Fig. 6C) indicated that the chromium was largely cell-associated and distributed throughout the cell. This is consistent with the approximate 40:60 distribution of the chromium between the cytoplasm and membrane fractions, when it is borne in mind that under the particulate methane monooxygenase (pMMO)-expressing conditions of these experiments, *M. capsulatus* Bath is expected to have intracellular as well as peripheral membranes (19).

The spatial distribution of chromium and a number of other elements (carbon, phosphorus, and oxygen) was also determined within whole *M. capsulatus* cells via EDX (Fig. S1 to S4). These results indicated that there was inhomogeneity in the distribution of all four elements, which appeared to correlate with structural features of the cells visible in the electron micrographs. Since these features showed elevated concentrations of chromium, phosphorus, and oxygen and decreased concentrations of carbon, they are consistent with the deposition of chromium phosphate (containing Cr, O, and P) associated with the cells. This is also consistent with the phosphorus and oxygen ligation of *M. capsulatus* cell-associated chromium(III) inferred from EXAFS data (20). The EDX analysis also indicated an increase in calcium associated with the cells that had been exposed to chromium (Fig. S1 to S4).

Surface analysis of samples of *M. capsulatus* cells treated with 20 mg of chromium(VI) liter^−1^ was performed, in comparison with chromium-untreated samples, by using X-ray photoelectron spectroscopy (XPS) (Fig. S5 and S6; Tables S1 and S2). While the depth of penetration of XPS into the sample is small (several nanometers), the areas of data acquisition were 400 μm in diameter. This is large compared to the cells (approximate diameter, 1 μm), and so these data show the properties of the surface of the whole sample rather than individual cells and subcellular structures seen by other techniques.

The carbon and oxygen X-ray photoelectron spectra of chromium(VI)-treated and chromium(VI)-untreated cells show features that are consistent with the presence of peptides, carbohydrates, lipids, and phosphate groups that are likely to be associated with the surfaces of the cells (Tables S1 and S2). The signals at binding energies of 400.1 eV [chromium(VI)-treated sample] and 399.8 eV [chromium(VI)-untreated sample] were attributed to N 1s, which is widely found in amino acids, and amino sugars (amino sugars are found in cell wall peptidoglycan, and amino acids are constituents of both peptidoglycan and proteins) (Fig. S5 and S6). No substantial differences in the spectra of carbon, oxygen, and nitrogen were observed between the chromium(VI)-treated and -untreated cells (Fig. S5 and S6; Tables S1 and S2).

Most notably, the small peak at a binding energy of 577.8 eV in the chromium(VI)-treated sample (Fig. S5A and E), which may be due to emission of photoelectrons from the 2p orbitals of chromium, was not substantially above the noise in the baseline of the spectrum. This indicates the presence of very little chromium on the surfaces of the cells, which is consistent with the results from analysis of the cell fractions that suggest that chromium is within the cells, associated with the membranes and cytoplasm.

## DISCUSSION

The results reported here indicate the capacity of *M. capsulatus* Bath to reduce chromium(VI) at concentrations up to several milligrams per liter or milligrams per gram, which are relevant to current contaminated groundwater and solid waste problems (7, 9–11). *M. capsulatus* Bath can, for example, take the concentration of hexavalent chromium from 3 mg liter^−1^ to below the level of detection of HPLC–ICP-MS (≤0.005 mg liter^−1^ in these experiments) within 48 h (Fig. 1). The nearly straight plots of *C*/*C*_0_ [ratio of supernatant chromium(VI) concentration at each time point to initial concentration] versus time (Fig. 1) at an initial chromium concentration of ≤10 mg liter^−1^ suggest near-zero-order kinetics at these low chromium concentrations. The rate of chromium(VI) removal during the first 48 h of incubation (Fig. S7) shows a dependence of chromium(VI) removal on the initial concentration, with a maximum at 20 mg liter^−1^ and a clear decline as the system becomes inhibited at higher concentrations of chromium(VI). In these experiments, total removal of detectable chromium(VI) was not achieved within 144 h from an initial concentration of 10 mg liter^−1^ or above. Although no significant removal of chromium(VI) was observed from an initial chromium concentration of 50 mg liter^−1^, reduction of a small proportion of added chromium(VI) by *M. capsulatus* must be possible at higher concentrations, since the cells from a culture exposed to 1,000 mg liter^−1^ of chromium(VI) contained chromium solely in the +3 oxidation state, although the amount of chromium(VI) reduced within the culture was not measured (20).

Previously, BLAST sequence similarity searches of the *M. capsulatus* Bath genome with representatives of three classes of chromium(VI) reductases and a chromate efflux pump were performed to identify proteins possibly involved in reduction of chromium(VI) (20). Since the genome of *M. trichosporium* OB3b (39), which does not reduce chromium(VI) (20), is now available, this analysis was repeated with both genomes in parallel (Table S3). The numbers of potential chromate reductases identified in the genomes of the two organisms are the same. Also, neither has a homologue of the chromate efflux pump. The chromate reductase ChrA of Pseudomonas putida (40) has a homologue in *M. trichosporium* OB3b but not in *M. capsulatus* Bath. The chromate reductase Fre of Escherichia coli (41) has only two homologues in *M. trichosporium* OB3b but three in *M. capsulatus* Bath. Among these, *M. capsulatus* alone has the gene encoding a putative F subunit of a Na^+^-translocating NADH-quinone reductase, which is part of a six-gene cluster encoding a possible transmembrane complex that transfers electrons between NADH and quinones (42) and that is missing from *M. trichosporium* OB3b. One possibility is that chromium(VI) reduction in *M. capsulatus* Bath is an adventitious activity of this complex, although it is also possible that other factors [such as the access of chromium(VI) to the reductase due to permeability properties] are the reason that *M. trichosporium* OB3b cells do not reduce chromium. The complexity of the reaction kinetics is consistent with a system in which the rate is influenced by multiple factors, such as enzyme activities, permeability, and toxicity.

Here, as in other studies, HAADF-STEM-EDX was used to study the distribution of metals in bacterial cells (33, 43, 44). The formation of cell-associated structures composed of precipitated metal ions has been observed previously, such as chromium-containing particles on the surface of a chromium(VI)-reducing Pseudomonas synxantha strain (45) and extracellular fibers and stalks of Fe(III) oxides formed by *Gallionella* (46). Chromium(VI) exposure of Shewanella oneidensis (35) and Desulfovibrio vulgaris (36) also results in deposition of chromium(III) in the form of precipitate on the surfaces of the cells, although intracellular chromium(II) has been observed under strictly anaerobic conditions in S. oneidensis and may be an intermediate in the conversion of chromium(VI) under conditions where the extracellular precipitated chromium(III) is produced by this organism (43). The cellular breakage and fractionation technique used here with *M. capsulatus* Bath showed the presence of all detectable chromium, after cell breakage, as chromium(III) within soluble cell-associated material and in association with membranes (or other small cell-derived particulate material with similar sedimentation properties). While the possibility of redistribution of chromium(III) after breakage of the cells cannot be excluded, the absence of extracellular precipitated chromium-containing material from TEM images, together with the shielding of chromium from XPS, give strong independent evidence of the intracellular location of the chromium(III) product. This intracellular chromium(III), which may be in the form of chromium(III) phosphate or chromium associated with organic phosphate groups, is visible to EDX and EELS in whole cells, as these are transmission techniques (results generated throughout the thickness of the sample).

*M. capsulatus* Bath was originally isolated from the Roman baths in Bath, United Kingdom, which are fed by water from a geothermal spring that is consistently low in toxic heavy metals, including chromium (<0.5 μg liter^−1^) (47). A strain of Methylomonas koyamae capable of removing chromium(VI) has also been isolated from river sediment with substantial heavy metal pollution (including chromium at up to 25 mg kg^−1^) (48). Hence, chromium(VI)-removing methanotrophs can be found in environments with greatly differing heavy metal contamination.

A number of studies have reported methane-driven chromium(VI) reduction by mixed communities of microorganisms (21, 49–51). These studies have attributed chromium(VI) reduction in such communities to the activities of nonmethanotrophs. The fact that a pure methanotroph strain is able to reduce chromium(VI) (20) indicates that methanotrophs may contribute to methane-driven chromium(VI) reduction in polymicrobial communities, including those which occur naturally in the environment. Studies of methane-driven reduction of chromium(VI) by mixed communities of microorganisms have shown that the bulk of the chromium in such polymicrobial systems is in the form of extracellular precipitate that is visible to XPS (21), in contrast to the intracellular location of the chromium(III) in pure cultures of *M. capsulatus* Bath.

Intracellular sequestration of chromium(III) by *M. capsulatus* Bath may help to reduce its bioavailability to other organisms and, since immobilization of chromium(III) has been linked to preventing its environmental reoxidation (3), likely make it less susceptible to reoxidation to chromium(VI). It is also possible that, under environmental conditions on a sufficiently long timescale, the cell-associated chromium(III) may be released into the environment.

While it is generally accepted that chromium(III) has lower toxicity than chromium(VI), previous studies have indicated detectable genotoxicity of chromium(III) to Escherichia coli (52), toxicity through generation of reactive oxygen species in Gram-positive and Gram-negative bacteria (53), and harmful morphological changes in Shewanella oneidensis (54). Hence, it is likely that the cells are substantially damaged by their uptake of chromium(III). They evidently maintain sufficient metabolism to allow the observed reduction and accumulation of chromium species observed, though it may be accumulation of the chromium(III) within the cells that causes decline in chromium(VI) removal as the externally applied chromium(VI) concentration is increased.

Previously, removal of chromium(III) by microorganisms has been largely attributed to adsorption to biomass; for example, biofilms of Bacillus subtilis were found to be more effective in immobilizing chromium(III) produced from reduction of chromium(VI) than planktonic cells, possibly owing to adsorption to extrapolymeric substances within the biofilm (55). The results with *M. capsulatus* suggest a rather different pattern, where uptake of external chromium(III) into the interior of the cell (cytoplasm and membranes) is an active process. Since most studies of chromium(VI) bioremediation have not investigated what happens if chromium(III) is added, it may be that active uptake of chromium(III) is found in other microorganisms also. The presence of chromium(III) to a substantial extent in soluble form in the cytoplasm fraction of *M. capsulatus* is consistent with observations that organic acids, amino acids, and other small biomolecules can maintain chromium(III) in soluble form in the presence of macromolecular biomass (56).

In order to establish the pathway of electrons between methane and chromium(VI), it will be necessary to identify the enzymes involved. Additionally, the effects of the availability of copper and lanthanides (which control the expression of different forms of methane monooxygenase, respectively) (30, 57–59), may offer a means of studying the pathway of electrons into chromium(VI) reduction.

Work in recent years has highlighted the key environmental role played in the environmental cycling of metals by aerobic methanotrophs (30), which show a much greater diversity than was previously realized (16). This study has demonstrated that one member of this important group of organisms shows a novel pattern of interaction with chromium species that may make it suitable for applications in bioremediation of chromium species and open the possibility for a role for methanotrophs in transformation and bioavailability of chromium species in the environment.

## MATERIALS AND METHODS

### Bacterial strains and growth conditions.

The methanotrophic bacterium *M. capsulatus* Bath was grown aerobically at 45°C in sterile nitrate mineral salts (NMS) medium with shaking (180 rpm) or NMS agar plates inside airtight jars, as described previously (60). Methanotrophs such as *M. capsulatus* Bath have a “copper switch” that controls the expression of the membrane-associated copper-dependent particulate methane monooxygenase (pMMO) versus the cytoplasmic iron-dependent soluble methane monooxygenase (sMMO) on the basis of the copper-to-biomass ratio of the culture (57, 61). In our hands, flask cultures grown at a copper concentration of 0.4 μM in the NMS used here do not attain the cell density needed to express sMMO, and so the experiments described were performed under pMMO-expressing conditions. Chromium(VI) bioremediation experiments were performed in 50-ml liquid cultures in 250-ml conical Quickfit flasks capped with Suba-Seals (Sigma-Aldrich). Plates were incubated in a 1:4 (vol/vol) methane-air mixture. To add methane to flasks, 50 ml of headspace gas was removed, after which 60 ml of methane was added (60). Liquid cultures (50 ml) were grown to late logarithmic phase (OD_600_ of 0.6 to 0.9), which typically took 40 to 48 h. Chromium species were added from filter-sterilized stock solutions containing 1,000 mg liter^−1^ of Cr [K_2_CrO_4_ for Cr(VI), and Cr(NO_3_)_3_ for Cr(III)] to the concentrations stated for each experiment.

### Quantitation and characterization of chromium species.

Separation and quantification of aqueous chromium(VI) and chromium(III) were achieved via ion exchange HPLC coupled to ICP-MS, as follows. An aliquot of the sample (20 μl) was injected by using a PerkinElmer LC Flexar autosampler into a PerkinElmer Flexar HPLC pump attached to a Hamilton PRP-X100 column, 4.6 by 250 mm, and coupled to a PerkinElmer ICP-MS NexION 350X instrument. The column flow rate was 1.2 ml min^−1^; the mobile phase was 0.5 mmol liter^−1^ EDTA disodium salt (Na_2_-EDTA) containing nitric acid (70% [wt/wt], 0.875 ml per liter of solution); aqueous ammonia was added to adjust the pH to 7. The limits of detection were 0.01 mg liter^−1^ for chromium(III) and 0.005 mg liter^−1^ for chromium(VI).

### Fractionation of cultures and cells.

Aliquots (5 ml) of cultures exposed to chromium(VI) or chromium(III) species as detailed above were collected at intervals and centrifuged (11,000 × *g*; 10 min; room temperature) to remove cells and other debris. The remaining culture supernatant was analyzed via HPLC–ICP-MS as detailed above.

Cells were fractionated via a modification of a published method (62), as follows. The whole process was performed at 0 to 4°C, to minimize sample degradation. Harvested cell pellets from 5-ml samples of culture were washed with 5 ml of 25 mM MOPS (morpholinepropanesulfonic acid, pH 7), centrifuged (11,000 × *g*; 10 min), and resuspended in 5 ml of the same buffer. The suspension was passed twice through a French pressure cell (8.2 MPa) in order to break the cell walls. The suspension of broken cells was centrifuged (3,000 × *g*, twice for 2 min each) to remove unbroken cells before being centrifuged (27,000 × *g*, 20 min) to sediment cell wall fragments and any other large broken cell fragments. The pellet was washed twice by resuspension in 25 mM MOPS (pH 7) and then resuspended in the same buffer; the resulting fraction was used as the cell wall fraction. The supernatant from the first centrifugation after cell breakage, which contained the cytoplasm and membrane fragments, was centrifuged again (27,000 × *g*, 20 min) to remove remaining large particulate material. The resulting supernatant was used as the cytoplasm-membrane fraction. When cytoplasm and membranes were analyzed separately, this fraction was further separated via ultracentrifugation at 105,000 × *g* for 60 min. The pellet was washed in 25 mM MOPS (pH 7), ultracentrifuged again under the same conditions, and resuspended in the same buffer to give the cell membrane fraction. The supernatant from the first ultracentrifugation was centrifuged again under the same conditions to remove remaining membranous material, to give the cytoplasm fraction.

### Imaging and surface analysis.

For electron microscopy, cells from samples (5 ml) of chromium(VI)-exposed cultures and control cultures without chromium were pelleted by centrifugation (11,000 × *g*; 10 min; room temperature) and washed under the same conditions with 0.1 M sodium phosphate buffer (pH 7.4). The specimens were then fixed in 3% glutaraldehyde in the same buffer overnight at room temperature and washed again under the same conditions in the same buffer. Secondary fixation was carried out in 1% (wt/vol) aqueous osmium tetroxide for 1 h at room temperature followed by the same washing procedure. Fixed cells were dehydrated through a graded series of ethanol dehydration steps (75, 95 and 100% [vol/vol]) and then placed in a 50/50 (vol/vol) mixture of ethanol and hexamethyldisilazane followed by 100% hexamethyldisilazane. The specimens were then allowed to air dry overnight. A small portion of the fixed sample was crushed and dispersed in methanol, with a drop placed on a holey carbon-coated copper grid (Agar Scientific). Transmission electron microscopy (TEM) was conducted on an FEI Titan^3^ Themis G2 instrument operating at 300 kV and fitted with 4 EDX silicon drift detectors, a Gatan OneView charge-coupled device (CCD) camera, and a Gatan GIF quantum ER 965 imaging filter for electron energy loss spectroscopy (EELS). Energy-dispersive X-ray (EDX) spectroscopy and mapping were undertaken using Bruker Esprit v1.9 software and a high-angle annular dark-field (HAADF) scanning TEM (STEM) detector.

For thin-section analysis, after the ethanol dehydration steps, the cells were embedded in EMbed 812 epoxy resin and cut into thin sections (90 nm) using a diamond knife on a Reichert Ultracut S ultramicrotome. The sections were supported on copper grids and coated with carbon. The samples were examined in an FEI Tecnai F20 field emission gun (FEG) transmission electron microscope operating at 200 kV and fitted with a Gatan Orius SC600A CCD camera, an Oxford Instruments XMax SDD energy-dispersive X-ray (EDX) detector, and a HAADF-STEM detector.

XPS measurements were made on a Kratos Supra photoelectron spectrometer at 10 kV and 20 mA using a monochromatic Al K (alpha) X-ray source (1,486.6 eV). The takeoff angle was fixed at 90°. For each sample, the data were collected from three randomly selected locations, and the area corresponding to each acquisition was 400 μm in diameter. Each analysis consisted of a wide survey scan (pass energy, 160 eV; 1.0-eV step size), and high-resolution scan (pass energy, 20 eV; 0.1-eV step size) for identification of component species. The binding energies of the peaks were determined using the C 1s peak at 284.5 eV. The software Casa XPS 2.3.17 was used to fit the XPS spectrum peaks. No constraint was applied to the initial binding energy values, and the full width at half maximum (FWHM) was kept constant for the carbon contributions in a particular spectrum.

### Bioinformatics.

BLAST searches of the proteins encoded by the *M. capsulatus* Bath and *M. trichosporium* OB3b genomes were conducted via the IMG platform (63).

## Supplementary Material

Supplemental file 1

## References

[1] Beukes JP, Du Preez SP, van Zyl PG, Paktunc D, Fabritius T, Päätalo M, Cramer M 2017 Review of Cr(VI) environmental practices in the chromite mining and smelting industry—relevance to development of the Ring of Fire, Canada. J Clean Prod 165:874–889. doi:10.1016/j.jclepro.2017.07.176.

[2] Coetzee JJ, Bansal N, Chirwa EMN 2020 Chromium in environment, its toxic effect from chromite-mining and ferrochrome industries, and its possible bioremediation. Expo Health 12:51–62. doi:10.1007/s12403-018-0284-z.

[3] Varadharajan C, Beller HR, Bill M, Brodie EL, Conrad ME, Han R, Irwin C, Larsen JT, Lim HC, Molins S, Steefel CI, Van Hise A, Yang L, Nico PS 2017 Reoxidation of chromium(III) products formed under different biogeochemical regimes. Environ Sci Technol 51:4918–4927. doi:10.1021/acs.est.6b06044. 28365989

[4] Hausladen DM, Fendorf S 2017 Hexavalent chromium generation within naturally structured soils and sediments. Environ Sci Technol 51:2058–2067. doi:10.1021/acs.est.6b04039. 28084730

[5] Wadhawan AR, Stone AT, Bouwer EJ 2013 Biogeochemical controls on hexavalent chromium formation in estuarine sediments. Environ Sci Technol 47:8220–8228. doi:10.1021/es401159b. 23802856

[6] Duckworth OW, Akafia MM, Andrews MY, Bargar JR 2014 Siderophore-promoted dissolution of chromium from hydroxide minerals. Environ Sci Process Impacts 16:1348–1359. doi:10.1039/c3em00717k. 24683601

[7] Hausladen DM, Alexander-Ozinskas A, McClain C, Fendorf S 2018 Hexavalent chromium sources and distribution in California groundwater. Environ Sci Technol 52:8242–8251. doi:10.1021/acs.est.7b06627. 29949365

[8] Vengosh A, Coyte R, Karr J, Harkness JS, Kondash AJ, Ruhl LS, Merola RB, Dywer GS 2016 Origin of hexavalent chromium in drinking water wells from the Piedmont aquifers of North Carolina. Environ Sci Technol Lett 3:409–414. doi:10.1021/acs.estlett.6b00342.

[9] Agarwal AK, Singh AP, Gupta T, Agarwal RA, Sharma N, Rajput P, Pandey SK, Ateeq B 2018 Mutagenicity and cytotoxicity of particulate matter emitted from biodiesel-fueled engines. Environ Sci Technol 52:14496–14507. doi:10.1021/acs.est.8b03345. 30512948

[10] Dhal B, Thatoi HN, Das NN, Pandey BD 2013 Chemical and microbial remediation of hexavalent chromium from contaminated soil and mining/metallurgical solid waste: a review. J Hazard Mater 250-251:272–291. doi:10.1016/j.jhazmat.2013.01.048. 23467183

[11] Jobby R, Jha P, Yadav AK, Desai N 2018 Biosorption and biotransformation of hexavalent chromium [Cr(VI)]: a comprehensive review. Chemosphere 207:255–266. doi:10.1016/j.chemosphere.2018.05.050. 29803157

[12] Cervantes C, Campos-García J, Devars S, Gutiérrez-Corona F, Loza-Tavera H, Torres-Guzmán JC, Moreno-Sánchez R 2001 Interactions of chromium with microorganisms and plants. FEMS Microbiol Rev 25:335–347. doi:10.1111/j.1574-6976.2001.tb00581.x. 11348688

[13] Zhang HK, Lu H, Wang J, Zhou JT, Sui M 2014 Cr(VI) reduction and Cr(III) immobilization by acinetobacter sp. HK-1 with the assistance of a novel quinone/graphene oxide composite. Environ Sci Technol 48:12876–12885. doi:10.1021/es5039084. 25296002

[14] Risso C, Choudhary S, Johannessen A, Silverman J 2018 Methanotrophy goes commercial: challenges, opportunities, and brief history, p 293–298. *In* Kalyuzhnaya MG, Xing X-H (ed), Methane biocatalysis: paving the way to sustainability. Springer International Publishing, Cham, Switzerland.

[15] Jiang H, Chen Y, Jiang P, Zhang C, Smith TJ, Murrell JC, Xing X-H 2010 Methanotrophs: multifunctional bacteria with promising applications in environmental bioengineering. Biochem Eng J 49:277–288. doi:10.1016/j.bej.2010.01.003.

[16] Smith TJ, Murrell JC 2008 Methanotrophy/methane oxidation, p 293–298. *In* Encyclopedia of industrial microbiology. Wiley, New York, NY.

[17] Strong PJ, Kalyuzhnaya M, Silverman J, Clarke WP 2016 A methanotroph-based biorefinery: potential scenarios for generating multiple products from a single fermentation. Bioresour Technol 215:314–323. doi:10.1016/j.biortech.2016.04.099. 27146469

[18] Kwon M, Ho A, Yoon S 2019 Novel approaches and reasons to isolate methanotrophic bacteria with biotechnological potentials: recent achievements and perspectives. Appl Microbiol Biotechnol 103:1–8. doi:10.1007/s00253-018-9435-1. 30315351

[19] Hanson RS, Hanson TE 1996 Methanotrophic bacteria. Microbiol Rev 60:439–471. doi:10.1128/MMBR.60.2.439-471.1996. 8801441 PMC239451

[20] Al Hasin A, Gurman SJ, Murphy LM, Perry A, Smith TJ, Gardiner PHE 2010 Remediation of chromium(VI) by a methane-oxidizing bacterium. Environ Sci Technol 44:400–405. doi:10.1021/es901723c. 20039753

[21] Lai C-Y, Zhong L, Zhang Y, Chen J-X, Wen L-L, Shi L-D, Sun Y-P, Ma F, Rittmann BE, Zhou C, Tang Y, Zheng P, Zhao H-P 2016 Bioreduction of chromate in a methane-based membrane biofilm reactor. Environ Sci Technol 50:5832–5839. doi:10.1021/acs.est.5b06177. 27161770

[22] Semrau JD, DiSpirito AA, Obulisamy PK, Kang-Yun CS 2020 Methanobactin from methanotrophs: genetics, structure, function and potential applications. FEMS Microbiol Lett 367:1–13. doi:10.1093/femsle/fnaa045. 32166327

[23] El Ghazouani A, Baslé A, Firbank SJ, Knapp CW, Gray J, Graham DW, Dennison C 2011 Copper-binding properties and structures of methanobactins from *Methylosinus trichosporium* OB3b. Inorg Chem 50:1378–1391. doi:10.1021/ic101965j. 21254756

[24] Vorobev A, Jagadevan S, Baral BS, DiSpirito AA, Freemeier BC, Bergman BH, Bandow NL, Semrau JD 2013 Detoxification of mercury by methanobactin from *Methylosinus trichosporium* OB3b. Appl Environ Microbiol 79:5918–5926. doi:10.1128/AEM.01673-13. 23872554 PMC3811387

[25] Choi DW, Do YS, Zea CJ, McEllistrem MT, Lee SW, Semrau JD, Pohl NL, Kisting CJ, Scardino LL, Hartsel SC, Boyd ES, Geesey GG, Riedel TP, Shafe PH, Kranski KA, Tritsch JR, Antholine WE, DiSpirito AA 2006 Spectral and thermodynamic properties of Ag(I), Au(III), Cd(II), Co(II), Fe(III), Hg(II), Mn(II), Ni(II), Pb(II), U(IV), and Zn(II) binding by methanobactin from *Methylosinus trichosporium* OB3b. J Inorg Biochem 100:2150–2161. doi:10.1016/j.jinorgbio.2006.08.017. 17070918

[26] McCabe JW, Vangala R, Angel LA 2017 Binding selectivity of methanobactin from *Methylosinus trichosporium* OB3b for copper(I), silver(I), zinc(II), nickel(II), cobalt(II), manganese(II), lead(II), and iron(II). J Am Soc Mass Spectrom 28:2588–2601. doi:10.1007/s13361-017-1778-9. 28856622

[27] Baral BS, Bandow NL, Vorobev A, Freemeier BC, Bergman BH, Herdendorf TJ, Fuentes N, Ellias L, Turpin E, Semrau JD, DiSpirito AA 2014 Mercury binding by methanobactin from *Methylocystis* strain SB2. J Inorg Biochem 141:161–169. doi:10.1016/j.jinorgbio.2014.09.004. 25265378

[28] Yin X, Wang L, Zhang L, Chen H, Liang X, Lu X, DiSpirito AA, Semrau JD, Gu B 2020 Synergistic effects of a chalkophore, methanobactin, on microbial methylation of mercury. Appl Environ Microbiol 86:e00122-20. doi:10.1128/AEM.00122-20. 32220843 PMC7237777

[29] Boden R, Murrell JC 2011 Response to mercury(II) ions in *Methylococcus capsulatus* (Bath). FEMS Microbiol Lett 324:106–110. doi:10.1111/j.1574-6968.2011.02395.x. 22092810

[30] Semrau JD, DiSpirito AA, Gu W, Yoon S 2018 Metals and methanotrophy. Appl Environ Microbiol 84:e02289-17. doi:10.1128/AEM.02289-17. 29305514 PMC5835748

[31] Dassama LMK, Kenney GE, Rosenzweig AC 2017 Methanobactins: from genome to function. Metallomics 9:7–20. doi:10.1039/c6mt00208k. 27905614 PMC5269455

[32] Eswayah AS, Smith TJ, Scheinost AC, Hondow N, Gardiner PHE 2017 Microbial transformations of selenite by methane-oxidizing bacteria. Appl Microbiol Biotechnol 101:6713–6724. doi:10.1007/s00253-017-8380-8. 28646447 PMC5554269

[33] Eswayah AS, Hondow N, Scheinost AC, Merroun M, Romero-González M, Smith TJ, Gardiner PHE 2019 Methyl selenol as a precursor in selenite reduction to Se/S species by methane-oxidizing bacteria. Appl Environ Microbiol 85:e01379-19. doi:10.1128/AEM.01379-19. 31519658 PMC6821961

[34] Wang Y, Sevinc PC, Belchik SM, Fredrickson J, Shi L, Lu HP 2013 Single-cell imaging and spectroscopic analyses of Cr(VI) reduction on the surface of bacterial cells. Langmuir 29:950–956. doi:10.1021/la303779y. 23249294 PMC3671764

[35] Neal AL, Lowe K, Daulton TL, Jones-Meehan J, Little BJ 2002 Oxidation state of chromium associated with cell surfaces of *Shewanella oneidensis* during chromate reduction. Appl Surf Sci 202:150–159. doi:10.1016/S0169-4332(02)00550-0.

[36] Goulhen F, Gloter A, Guyot F, Bruschi M 2006 Cr(VI) detoxification by *Desulfovibrio vulgaris* strain Hildenborough: microbe-metal interactions studies. Appl Microbiol Biotechnol 71:892–897. doi:10.1007/s00253-005-0211-7. 16896506

[37] Daulton TL, Little BJ, Lowe K, Jones-Meehan J 2002 Electron energy loss spectroscopy techniques for the study of microbial chromium(VI) reduction. J Microbiol Methods 50:39–54. doi:10.1016/S0167-7012(02)00013-1. 11943357

[38] Chardin B, Dolla A, Chaspoul F, Fardeau M, Gallice P, Bruschi M 2003 Bioremediation of chromate: thermodynamic analysis of the effects of Cr(VI) on sulfate-reducing bacteria. Appl Microbiol Biotechnol 60:352–360. 10.1007/s00253-002-1091-8 12436319

[39] Stein LY, Yoon S, Semrau JD, DiSpirito AA, Crombie A, Murrell JC, Vuilleumier S, Kalyuzhnaya MG, Op den Camp HJM, Bringel F, Bruce D, Cheng JF, Copeland A, Goodwin L, Han S, Hauser L, Jetten MSM, Lajus A, Land ML, Lapidus A, Lucas S, Medigue C, Pitluck S, Woyke T, Zeytun A, Klotz MG 2010 Genome sequence of the obligate methanotroph *Methylosinus trichosporium* strain OB3b. J Bacteriol 192:6497–6498. doi:10.1128/JB.01144-10. 20952571 PMC3008524

[40] Ackerley DF, Gonzalez CF, Park CH, Blake R, Keyhan M, Matin A 2004 Chromate-reducing properties of soluble flavoproteins from *Pseudomonas putida* and *Escherichia coli*. Appl Environ Microbiol 70:873–882. doi:10.1128/aem.70.2.873-882.2004. 14766567 PMC348923

[41] Puzon GJ, Petersen JN, Roberts AG, Kramer DM, Xun L 2002 A bacterial flavin reductase system reduces chromate to a soluble chromium(III)-NAD^+^ complex. Biochem Biophys Res Commun 294:76–81. doi:10.1016/S0006-291X(02)00438-2. 12054743

[42] Barquera B, Hellwig P, Zhou W, Morgan JE, Häse CC, Gosink KK, Nilges M, Bruesehoff PJ, Roth A, Lancaster CRD, Gennis RB 2002 Purification and characterization of the recombinant Na^+^-translocating NADH:quinone oxidoreductase from *Vibrio cholerae*. Biochemistry 41:3781–3789. doi:10.1021/bi011873o. 11888296

[43] Daulton TL, Little BJ, Jones-Meehan J, Blom DA, Allard LF 2007 Microbial reduction of chromium from the hexavalent to divalent state. Geochim Cosmochim Acta 71:556–565. doi:10.1016/j.gca.2006.10.007.

[44] Villagrasa E, Ballesteros B, Obiol A, Millach L, Esteve I, Solé A 2020 Multi-approach analysis to assess the chromium(III) immobilization by *Ochrobactrum anthropi* DE2010. Chemosphere 238:124663. doi:10.1016/j.chemosphere.2019.124663. 31472343

[45] McLean JS, Beveridge TJ, Phipps D 2000 Isolation and characterization of a chromium-reducing bacterium from a chromated copper arsenate-contaminated site. Environ Microbiol 2:611–619. doi:10.1046/j.1462-2920.2000.00143.x. 11214794

[46] Hallberg R, Ferris FG 2004 Biomineralization by *Gallionella*. Geomicrobiol J 21:325–330. doi:10.1080/01490450490454001.

[47] Edmunds WM, Darling WG, Purtschert R, Corcho Alvarado JA 2014 Noble gas, CFC and other geochemical evidence for the age and origin of the Bath thermal waters, UK. Appl Geochem 40:155–163. doi:10.1016/j.apgeochem.2013.10.007.

[48] Challa S, Smith TJ 2020 Isolation of a methane‐oxidizing bacterium that bioremediates hexavalent chromium from a formerly industrialized suburban river. Lett Appl Microbiol 71:287–293. doi:10.1111/lam.13330. 32470995

[49] Lv P, Zhong L, Dong Q, Yang S, Shen W, Zhu Q, Lai C, Luo A-C, Tang Y, Zhao H-P 2018 The effect of electron competition on chromate reduction using methane as electron donor. Environ Sci Pollut Res Int 25:6609–6618. doi:10.1007/s11356-017-0937-7. 29255986

[50] Lu YZ, Chen GJ, Bai YN, Fu L, Qin LP, Zeng RJ 2018 Chromium isotope fractionation during Cr(VI) reduction in a methane-based hollow-fiber membrane biofilm reactor. Water Res 130:263–270. doi:10.1016/j.watres.2017.11.045. 29241112

[51] Long M, Zhou C, Xia S, Guadiea A 2017 Concomitant Cr(VI) reduction and Cr(III) precipitation with nitrate in a methane/oxygen-based membrane biofilm reactor. Chem Eng J 315:58–66. doi:10.1016/j.cej.2017.01.018.

[52] Plaper A, Jenko-Brinovec Š, Premzl A, Kos J, Raspor P 2002 Genotoxicity of trivalent chromium in bacterial cells. Possible effects on DNA topology. Chem Res Toxicol 15:943–949. doi:10.1021/tx010096q. 12119005

[53] Fathima A, Rao JR 2018 Is Cr(III) toxic to bacteria: toxicity studies using *Bacillus subtilis* and *Escherichia coli* as model organism. Arch Microbiol 200:453–462. doi:10.1007/s00203-017-1444-4. 29189889

[54] Parker DL, Borer P, Bernier-Latmani R 2011 The response of *Shewanella oneidensis* MR-1 to Cr(III) toxicity differs from that to Cr(VI). Front Microbiol 2:1–14. doi:10.3389/fmicb.2011.00223. 22125549 PMC3221395

[55] Pan X, Liu Z, Chen Z, Cheng Y, Pan D, Shao J, Lin Z, Guan X 2014 Investigation of Cr(VI) reduction and Cr(III) immobilization mechanism by planktonic cells and biofilms of *Bacillus subtilis* ATCC-6633. Water Res 55:21–29. doi:10.1016/j.watres.2014.01.066. 24583840

[56] Cheng Y, Yan F, Huang F, Chu W, Pan D, Chen Z, Zheng J, Yu M, Lin Z, Wu Z 2010 Bioremediation of Cr(VI) and immobilization as Cr(III) by *Ochrobactrum anthropi*. Environ Sci Technol 44:6357–6363. doi:10.1021/es100198v. 20608725

[57] Stanley SH, Prior SD, Leak DJ, Dalton H 1983 Copper stress underlies the fundamental change in intracellular location of methane mono-oxygenase in methane-oxidizing organisms: studies in batch and continuous cultures. Biotechnol Lett 5:487–492. doi:10.1007/BF00132233.

[58] Gu W, Semrau JD 2017 Copper and cerium-regulated gene expression in *Methylosinus trichosporium* OB3b. Appl Microbiol Biotechnol 101:8499–8516. doi:10.1007/s00253-017-8572-2. 29032471

[59] Kao WC, Chen YR, Yi EC, Lee H, Tian Q, Wu KM, Tsai SF, Yu SSF, Chen YJ, Aebersold R, Chan SI 2004 Quantitative proteomic analysis of metabolic regulation by copper ions in *Methylococcus capsulatus* (Bath). J Biol Chem 279:51554–51560. doi:10.1074/jbc.M408013200. 15385566

[60] Smith TJ, Murrell JC 2011 Mutagenesis of soluble methane monooxygenase. Methods Enzymol 495:135–147. doi:10.1016/B978-0-12-386905-0.00009-7. 21419919

[61] Smith TJ, Murrell JC 2009 Methanotrophy/methane oxidation, p 293–298. *In* Schaechter M (ed), Encyclopedia of microbiology, 3rd ed Elsevier B.V, Amsterdam, The Netherlands.

[62] Smith TJ, Foster SJ 2006 Autolysins during sporulation of *Bacillus subtilis* 168. FEMS Microbiol Lett 157:141–147. doi:10.1111/j.1574-6968.1997.tb12765.x.

[63] Chen IMA, Chu K, Palaniappan K, Pillay M, Ratner A, Huang J, Huntemann M, Varghese N, White JR, Seshadri R, Smirnova T, Kirton E, Jungbluth SP, Woyke T, Eloe-Fadrosh EA, Ivanova NN, Kyrpides NC 2019 IMG/M v.5.0: an integrated data management and comparative analysis system for microbial genomes and microbiomes. Nucleic Acids Res 47:D666–D677. doi:10.1093/nar/gky901. 30289528 PMC6323987

