# Supplementary material for: Detoxification, Active Uptake, and Intracellular Accumulation of Chromium Species by a Methane-Oxidizing Bacterium
Source: Appl Environ Microbiol. 2021 Jan 4;87(2):e00947-20. doi: 10.1128/AEM.00947-20 (PMC7783347; doi:10.1128/AEM.00947-20)
Supplement: Supplemental file 1 [file AEM.00947-20-s0001.pdf]

Supplemental Material

Detoxification, active uptake, and intracellular accumulation of chromium species by a methane-oxidising bacterium

Salaheldeen Enbaia [a], Abdurrahman Eswayah [a], Nicole Hondow [b], Philip H. E. Gardiner [a] and Thomas J. Smith [*,a].

[a] Biomolecular Sciences Research Centre, Sheffield Hallam University, Howard Street, Sheffield S1 1WB, United Kingdom.

[b] School of Chemical and Process Engineering, University of Leeds, Leeds LS2 9JT, United Kingdom.

* To whom correspondence should be addressed: Tel. +44 (0) 114 225 3042. Email: t.j.smith@shu.ac.uk

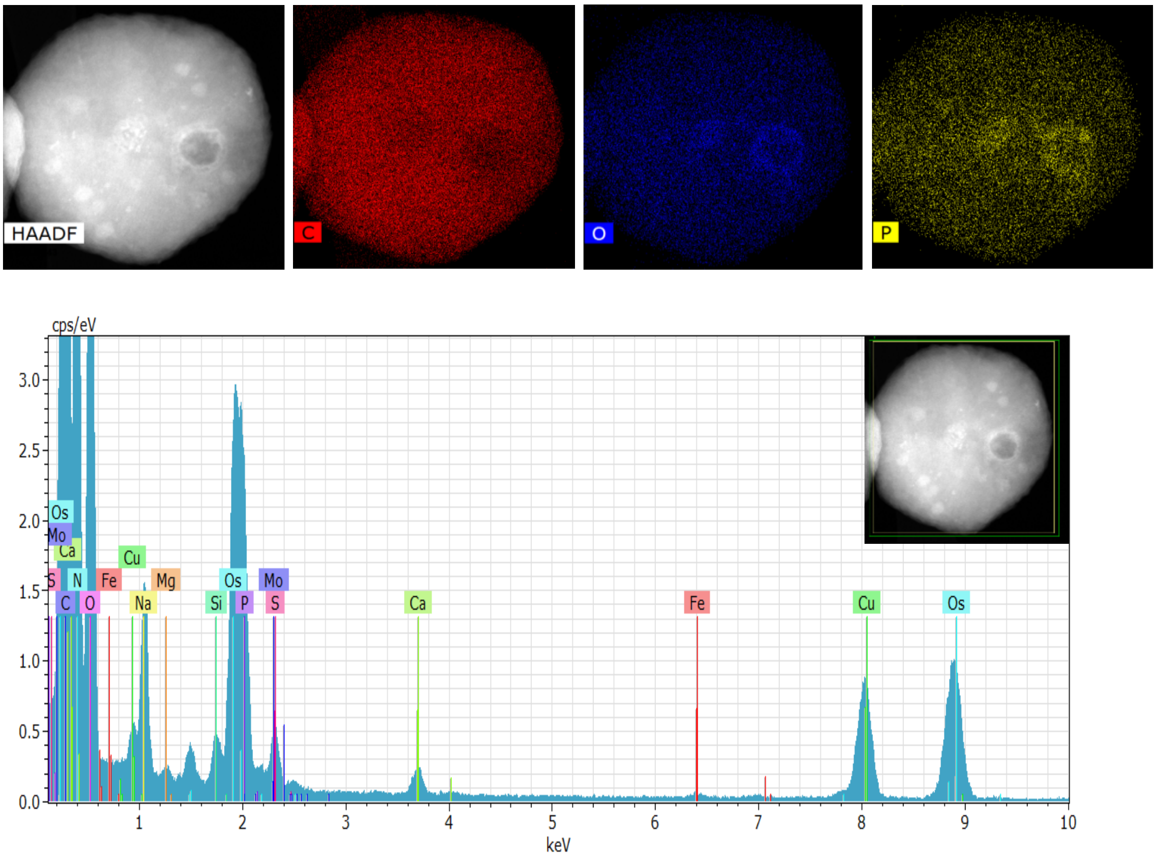

FIG S1 Electron micrographs with corresponding EDX spectra of whole *M. capsulatus* Bath cells, showing the distribution of carbon, oxygen and phosphorous after incubation of the culture in the presence of methane for 144 h at 45°C without added chromium. The EDX spectrum was generated from data collected from the area indicated by the box in the insert.

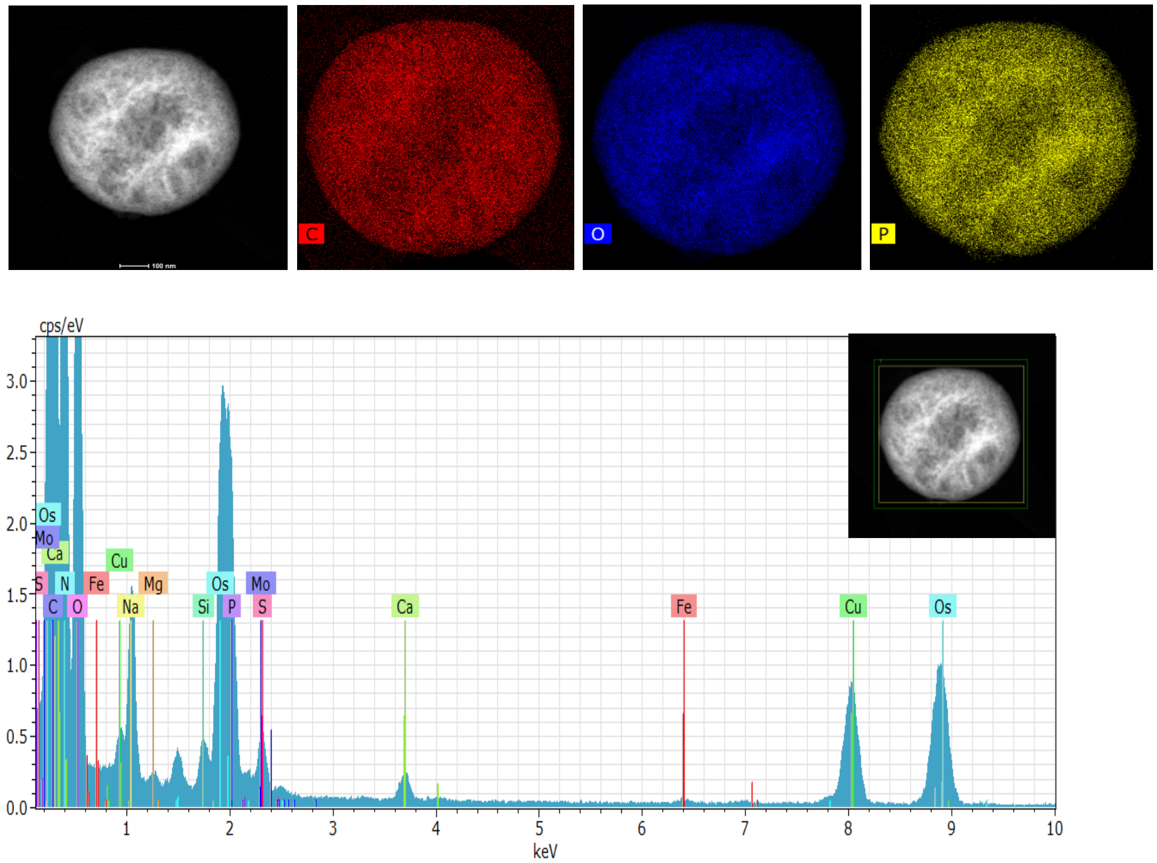

FIG S2 Electron micrographs showing the distribution of elements via EDX spectroscopy of whole *M. capsulatus* Bath cells, after incubation of the culture in the presence of methane for 144 h at 45°C without added chromium. The EDX spectrum was generated from data collected from the area indicated by the box in the insert. This figure shows a different area from the sample analysed in Fig. S1.

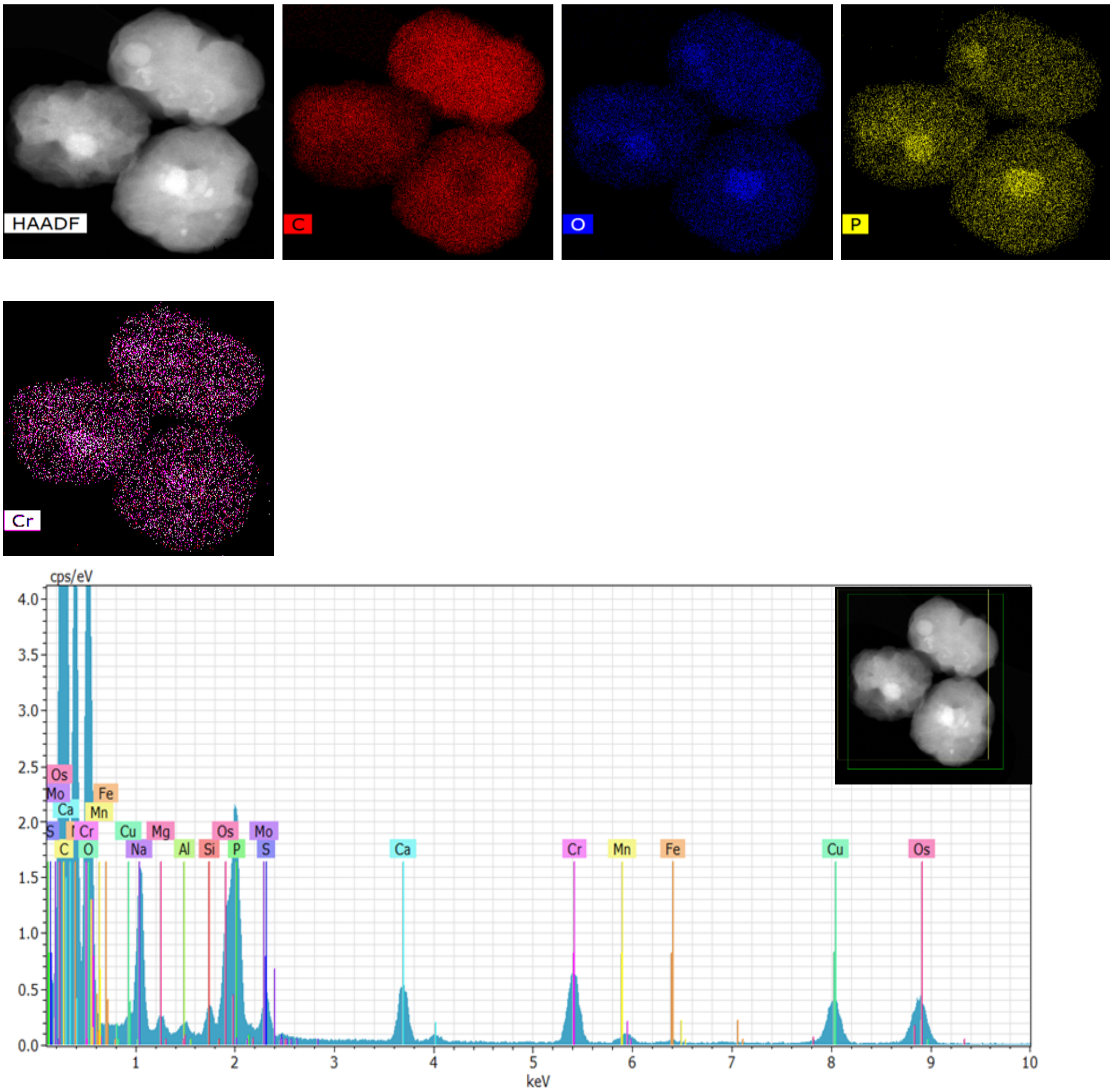

FIG S3 Electron micrographs with corresponding EDX spectra of whole *M. capsulatus* Bath cells, showing the distribution of carbon, oxygen and phosphorous after incubation of the culture in the presence of methane for 144 h at 45°C after addition of chromium (VI) to 20 mg L^{-1}. The EDX spectrum was generated from data collected from the area indicated by the box in the insert.

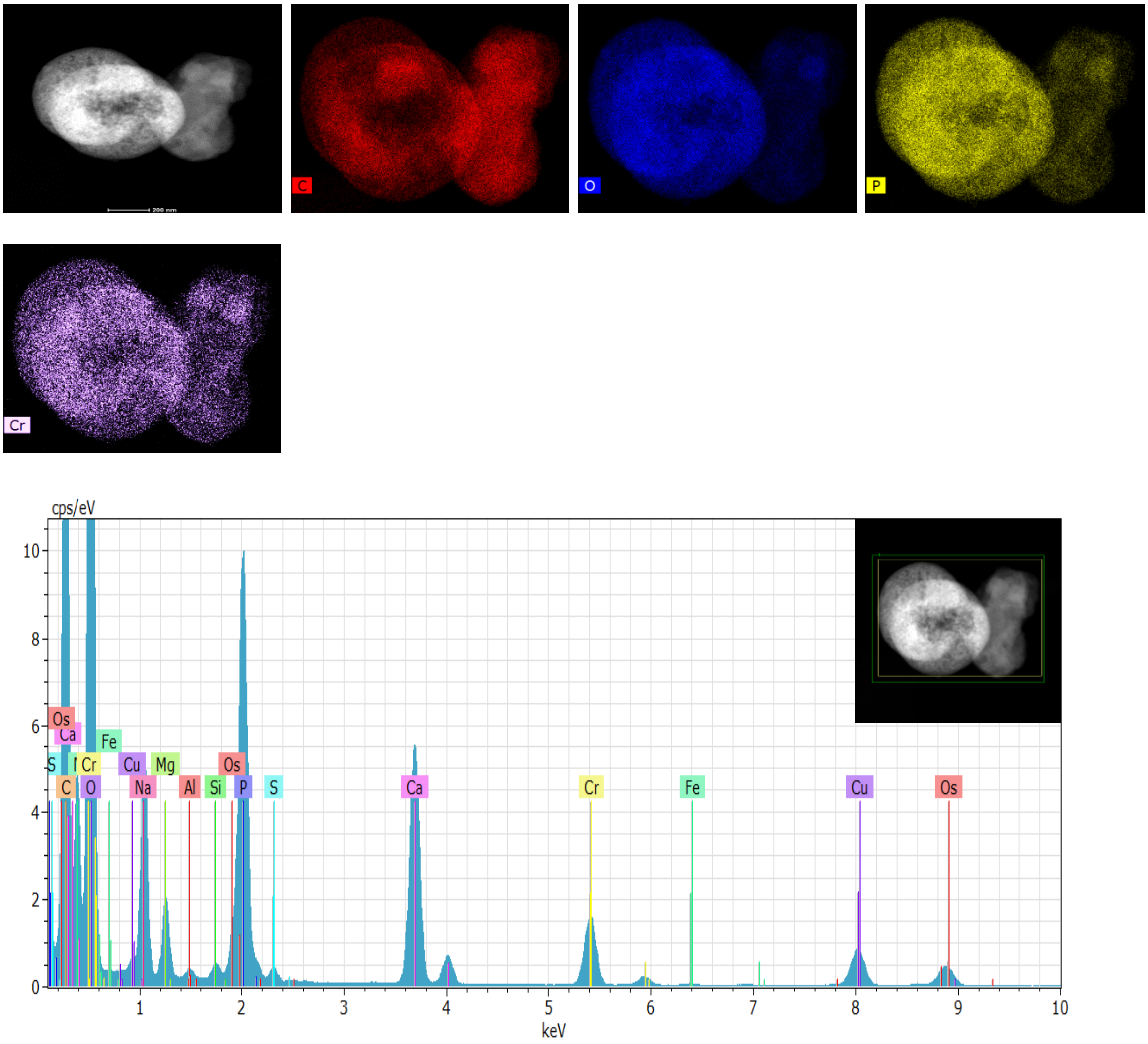

FIG S4 Electron micrographs showing the distribution of elements via EDX spectroscopy of whole *M. capsulatus* Bath cells, after incubation of the culture in the presence of methane for 144 h at 45°C after addition of chromium (VI) to 20 mg L^{-1}. The EDX spectrum was generated from data collected from the area indicated by the box in the insert. This figure shows a different area from the sample analysed in Fig. S3.

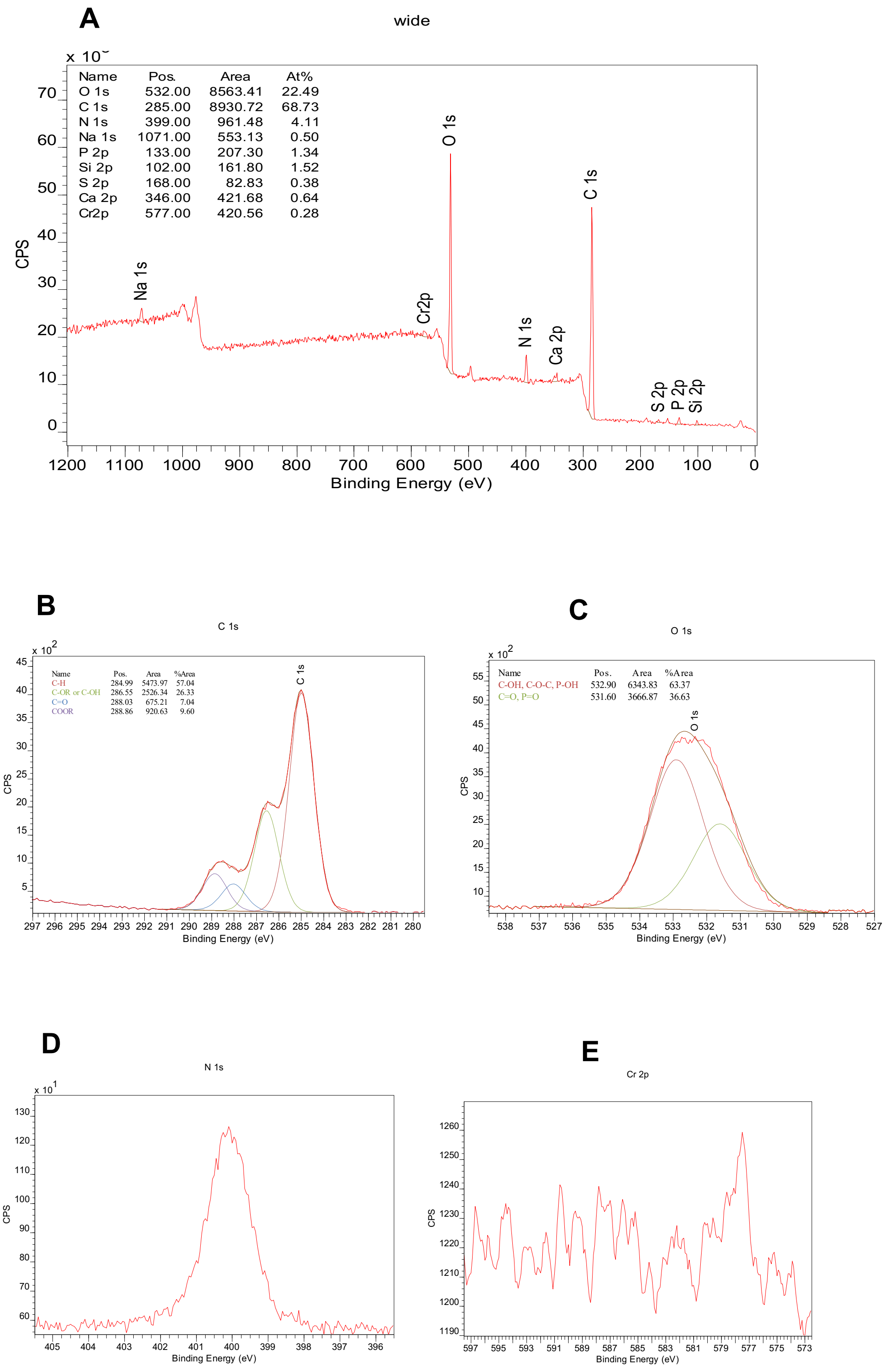

FIG S5 Wide scan X-ray photoelectron spectra of *M. capsulatus* Bath cells exposed to 20 mg L^{-1} of chromium (VI) for 144 h (A) and high resolution spectra for C 1s and O 1s are shown in B and C. The low resolution spectra for N 1s and Cr 2p are shown in D and E, respectively.

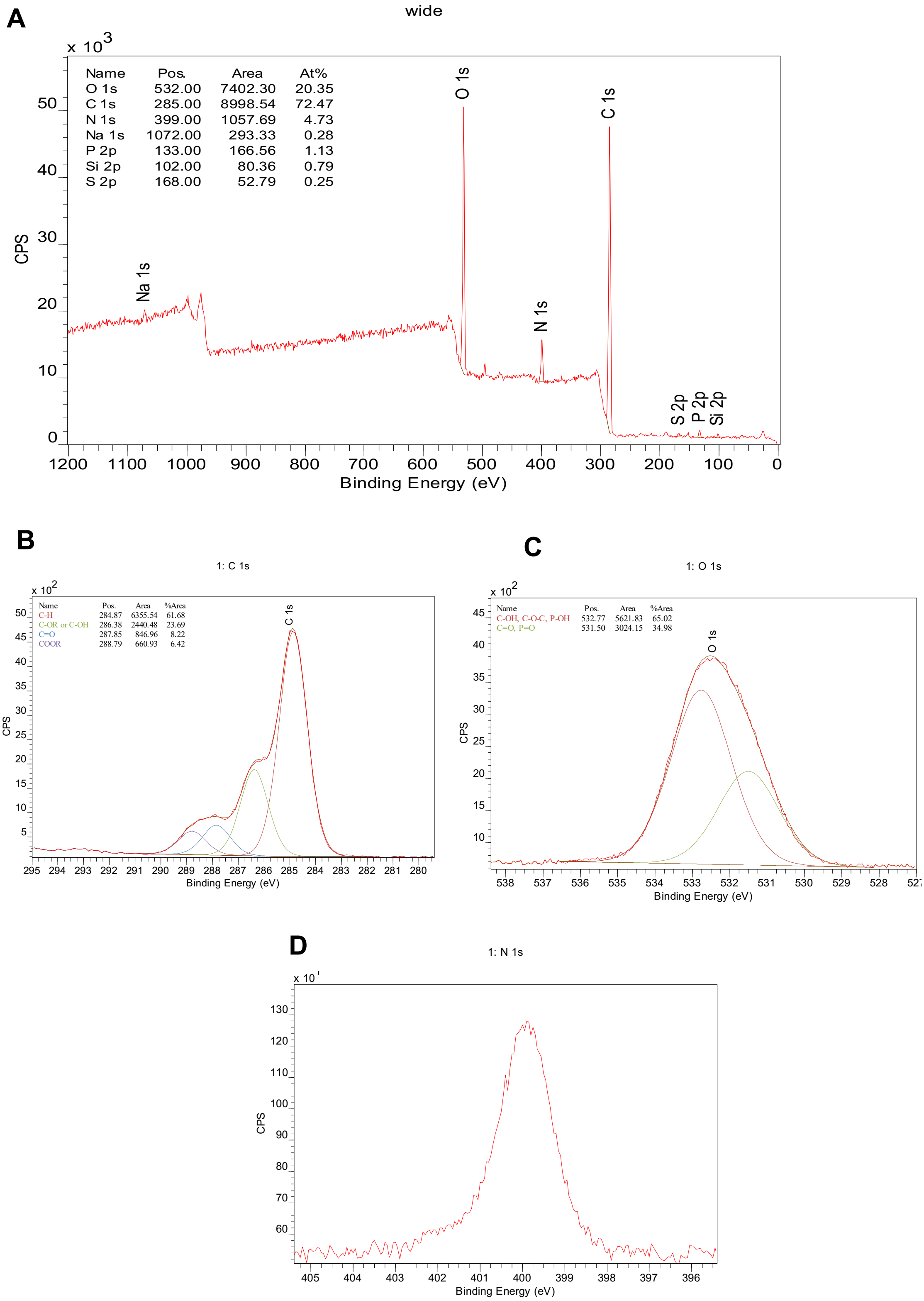

FIG S6 Wide scan X-ray photoelectron spectra of control sample to *M. capsulatus* Bath that was not exposed to chromium (A) and high resolution spectra for C 1s and O 1s are shown in B and C. The low resolution spectra for N 1s is shown in D.

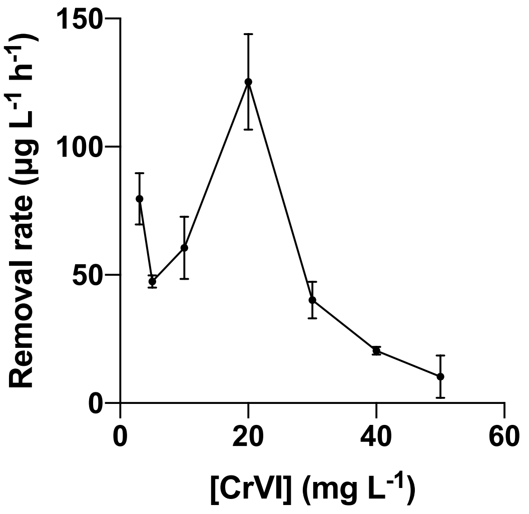
150
100
50
0
Removal rate (µg L-1 h-1)
0
20
40
60
[CrVI] (mg L-1)

FIG S7 Rate of removal of chromium (VI) by cultures of *M. capsulatus* Bath calculated from the fall in chromium (VI) concentration during the first 24 h of incubation. Error bars show standard deviations of triplicate biological replicates. This graph was constructed using the same data used to construct Fig. 1 in the main text.

TABLE S1. Results of curve-appropriate C 1s spectra of *Mc. capsulatus* Bath of (control sample compared with chromium-treated sample). (B. E. = binding energy).

Control sample			Chromium sample		
Name of group	B. E. (eV)	% Area	Name of group	B. E. (eV)	% Area
C - H	284.87	61.68	C - H	284.99	57.04
C- OR or C - OH	286.38	23.69	C- OR or C - OH	286.55	26.33
C = O	287.85	8.22	C = O	288.03	7.04
COOR	288.79	6.42	COOR	288.86	9.60

TABLE S2. Results of curve-appropriate O 1s spectra of *M. capsulatus* Bath of (control sample compared with chromium-treated sample). (B. E. = binding energy).

Control sample			Chromium sample		
Name of group	B. E. (eV)	% Area	Name of group	B. E. (eV)	% Area
C - H, C - O - C, P - OH	532.77	65.02	C - H, C - O - C, P - OH	532.90	63.37
C = O, P = O	531.50	34.98	C = O, P = O	531.60	36.63

TABLE S3. Chromium (VI) reductase and permease homologues derived from BLAST-P searches of the *M. capsulatus* Bath and *M. trichosporium* OB3b genomes.

**Query sequence** (accession number)	**Similar sequences** (E < 10^{-5})	
	***M. capsulatus* Bath**	***M. trichosporium* OB3b**
Fre chromate reductase of *Escherichia coli* (M74448)	Soluble methane monooxygenase reductase component (locus tag MCA1200)	Soluble methane monooxygenase reductase component (locus tag Ga0263880_112528)
	Oxygenase, putative (MCA2508)	Ferredoxin-NADP reductase (Ga0263880_113028)
	Na(+)-translocating NADH-quinone reductase subunit F (MCA2384)	
Nitroreductase NfsA of *E. coli* (BAA35562)	Nitroreductase family protein (MCA1372)	Nitroreductase (Ga0263880_114180)
Old Yellow Enzyme chromate reductase of *Thermus scotoductus* (CAP16804)	NADH-dependent flavin oxidoreductase, Oye family (MCA0639)	2,4-dienoyl-CoA reductase-like NADH-dependent reductase (Old Yellow Enzyme family) (Ga0263880_11482)
ChrR chromate reductase of *Pseudomonas putida* (Q93T20)	None	NAD(P)H-dependent FMN reductase (Ga0263880_113996)
ChrA chromate efflux pump of *Pseudomonas aeruginosa* plasmid pUM505 (M29034)	None	None
